# Prostate Cancer With Orbital Metastasis

**DOI:** 10.7759/cureus.80727

**Published:** 2025-03-17

**Authors:** Swathi Gopishetty, Lavi Singh, Mohammad Aqil, Abdullah Elhalis, Daniel Ezekwudo

**Affiliations:** 1 Hematology and Oncology, Corewell Health William Beaumont University Hospital, Royal Oak, USA; 2 Hematology and Oncology, Oakland University William Beaumont School of Medicine, Rochester, USA; 3 Hematology and Oncology, Michigan State University, East Lansing, USA

**Keywords:** androgen deprivation therapy, bone metastases, chemotherapy, elevated psa, metastatic disease, orbital metastasis, prostate cancer, vision loss, visual impairment

## Abstract

Prostate cancer (PCa) is one of the most commonly diagnosed cancers in men worldwide, with an increasing incidence due to advancements in detection methods. The majority of PCa cases are sporadic, though a small percentage is hereditary. PCa primarily metastasizes to the bones, followed by lymph nodes, liver, and thorax. However, metastasis to atypical sites, including the orbit, remains exceedingly rare. Orbital metastasis from PCa is associated with a poor prognosis and can lead to significant visual impairment. This case report describes a 71-year-old male patient with a history of hypertension, atrial fibrillation, and obstructive uropathy, who presented with diplopia and left-sided ptosis. Imaging revealed a left intraconal orbital mass with intracranial extension, leading to the diagnosis of metastatic prostate adenocarcinoma. Further evaluation revealed extensive bone metastases and retroperitoneal lymphadenopathy. The patient was diagnosed with high tumor burden and castration-naïve metastatic PCa and treated with a combination regimen of androgen deprivation therapy, docetaxel, and darolutamide, resulting in significant reduction in prostate-specific antigen levels. Despite initial success in lowering tumor burden, the patient experienced side effects, including a rash, leading to discontinuation of chemotherapy. This case underscores the importance of considering PCa in the differential diagnosis of orbital tumors, despite its rarity. Surgical resection and palliative radiation were employed to manage the orbital mass, and ongoing treatment with denosumab was initiated to address the extensive bone metastases. This case highlights the clinical challenges and treatment considerations in patients with atypical metastatic spread of PCa and emphasizes the need for a multidisciplinary approach in managing such complex presentations.

## Introduction

The second most frequent cancer diagnosis made in men worldwide is prostate cancer (PCa) [1]. While the mortality of men diagnosed with PCa is 1 in 8, the most common confounding variables include cardiovascular and cerebrovascular diseases and chronic obstructive pulmonary disease [2]. The risk of PCa increases with age, with the median age of diagnosis being above 60 years old. According to data obtained from the GLOBOCAN 2020 database, there were over 375,000 reported deaths related to PCa. Due to the improvements in PCa detection, the percentage of PCa survivors has increased [3]. Elevated levels of prostate-specific antigen (PSA) have been frequently used to detect PCa. That being said since PSA can be elevated in men without PCa, a tissue biopsy is utilized to confirm the detection of cancer, with digital rectal examinations (DRE) and magnetic resonance imaging (MRI) also being used [1,4]. The majority of PCa cases are sporadic, with about 5-15% of cases being attributed to hereditary factors [5]. 

The most common metastatic site of PCa is the bone, which is about 80-85%, followed by the lymph nodes, liver, and thorax [6]. Multiple metastatic sites can be involved in PCa patients (less frequently in bone metastasis). The proportion of men with multiple site involvement, such as lymph nodes, liver, thorax, brain, digestive system, retroperitoneum, kidney, and adrenal gland metastases, was 43.4%, 76.0%, 76.7%, 73.0%, 52.2%, 60.9%, and 76.4%, respectively [7]. Atypical site involvement is not very uncommon in PCa. Orbital metastasis remains exceedingly rare, with studies estimating that only a small percentage of cases involve orbital spread [8]. This rarity makes orbital metastasis from PCa a unique and challenging presentation, often associated with poor prognosis and significant visual impairment [9]. 

This report educates about the rare occurrence of prostate adenocarcinoma metastasizing into the orbits and the treatment involved for the associated visual deficits, which included an orbital cranial tumor resection. Correctly identifying the origin of the tumor, in this case, prostatic, will help improve visual outcomes in patients with similar complications. 

## Case presentation

**Figure 1:**
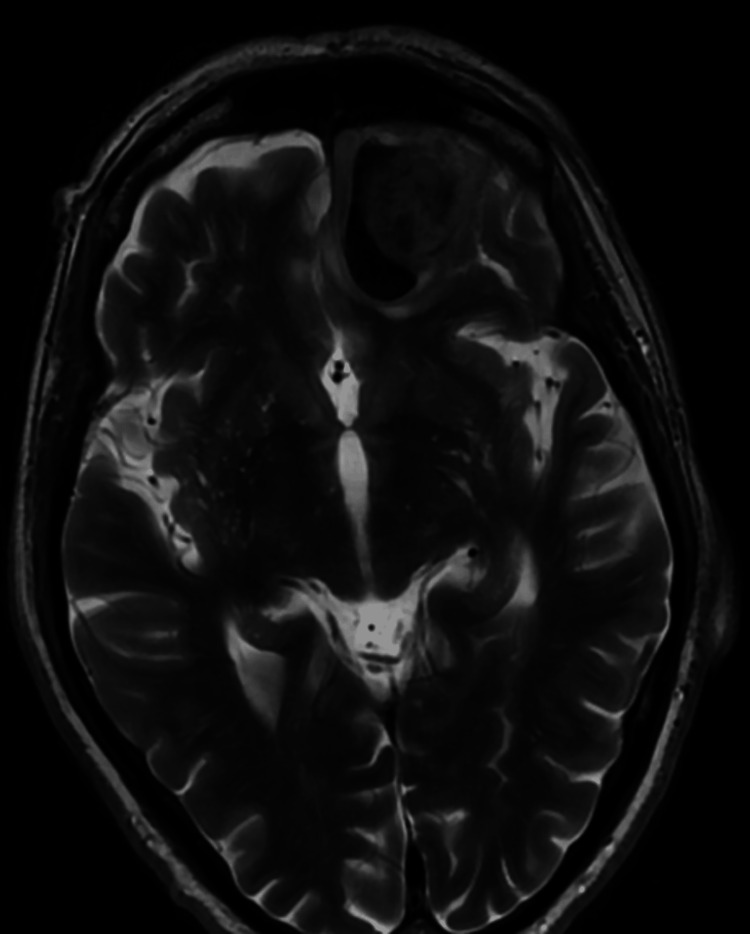
MRI brain axial view

**Figure 2:**
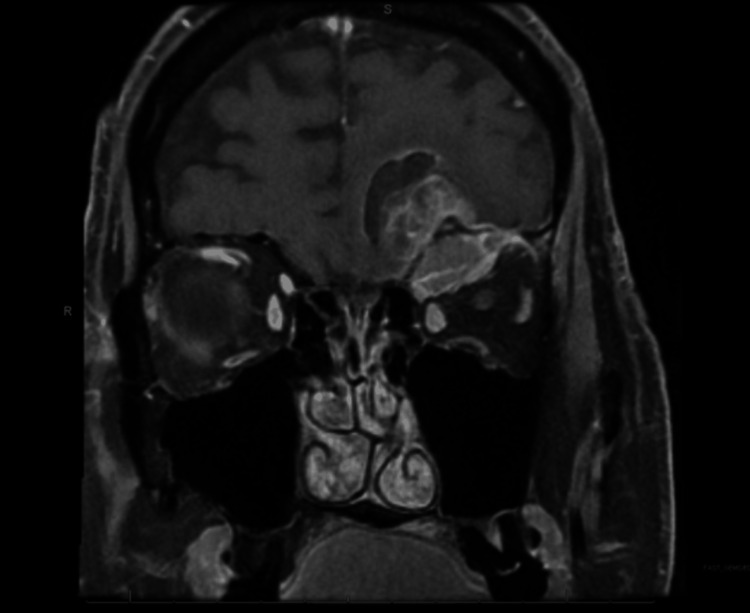
MRI brain and orbit coronal view

A 71-year-old man presented with a history of hypertension, atrial fibrillation, and ureteral stricture resulting in obstructive uropathy status post-stent placement. He initially presented to an ophthalmology clinic with the complaint of diplopia and left-sided ptosis. On clinical examination, the patient was in good general condition. His visual acuity in both eyes was abnormal. MRI of the brain and orbits (Figures 1, 2) revealed a heterogeneously enhancing left intraconal mass within the superior aspect of the orbit with extraconal/intracranial extension through the superior orbital bone into the inferior left frontal lobe measuring up to 3.0 cm. There was also surrounding mass effect without any midline shift. There was minimal inferior displacement of the left superior rectus muscle, orbital globe, and suprapubic muscle without proptosis. The optic nerves were intact. 

**Figure 3:**
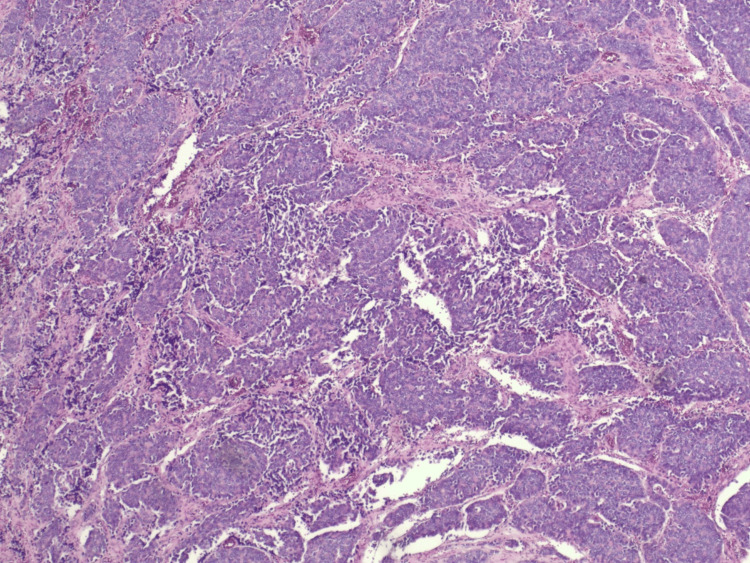
H&E-stained section showing metastatic carcinoma with tumor cells arranged in sheets and nests, exhibiting a high nuclear-to-cytoplasmic ratio and prominent nucleoli H&E: Hematoxylin and Eosin

**Figure 4:**
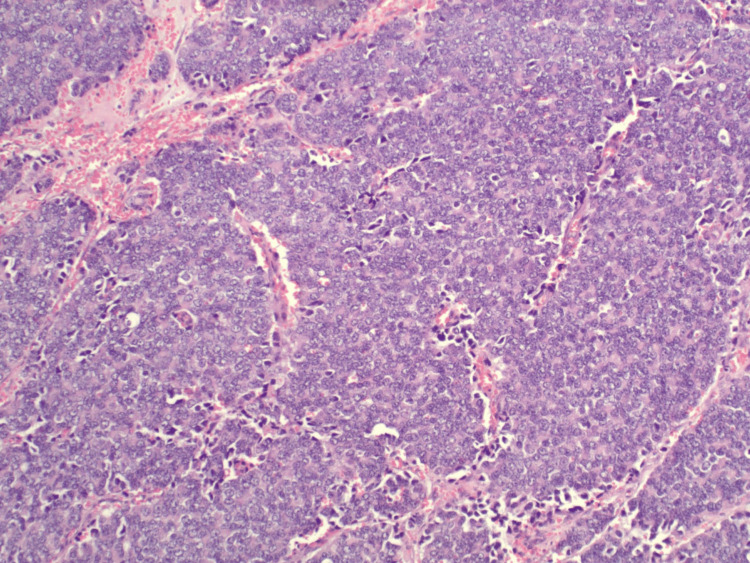
H&E-stained section showing metastatic carcinoma with areas of necrosis and stromal reaction, highlighting infiltrative tumor architecture H&E: Hematoxylin and Eosin

He subsequently underwent orbital cranial tumor resection and his pathology showed metastatic poorly differentiated adenocarcinoma most consistent with prostate origin (Figures 3, 4). 

**Figure 5:**
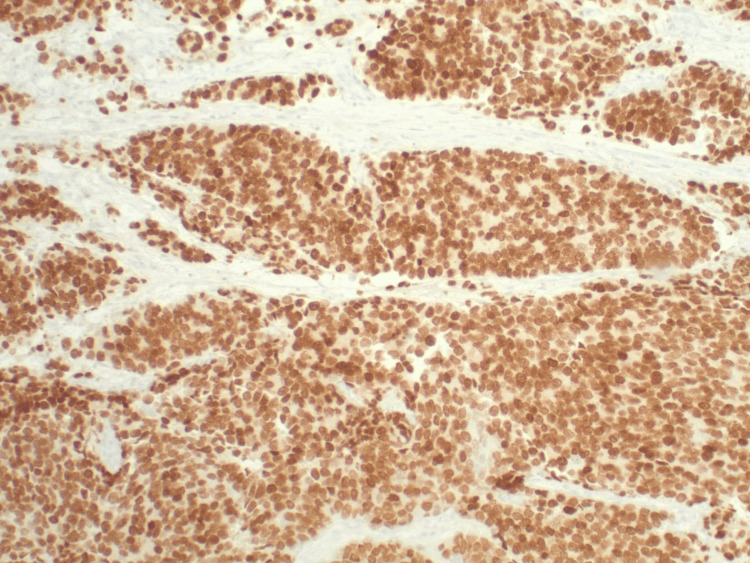
Cells with a markedly elevated nuclear/cytoplasmic ratio and prominent nucleoli.

Figure 5 shows cells that have a markedly elevated nuclear/cytoplasmic ratio and prominent nucleoli. NKX3.1 immunohistochemistry exhibits diffuse nuclear staining confirming prostatic origin. PAP (not shown) is also positive. 

**Figure 6:**
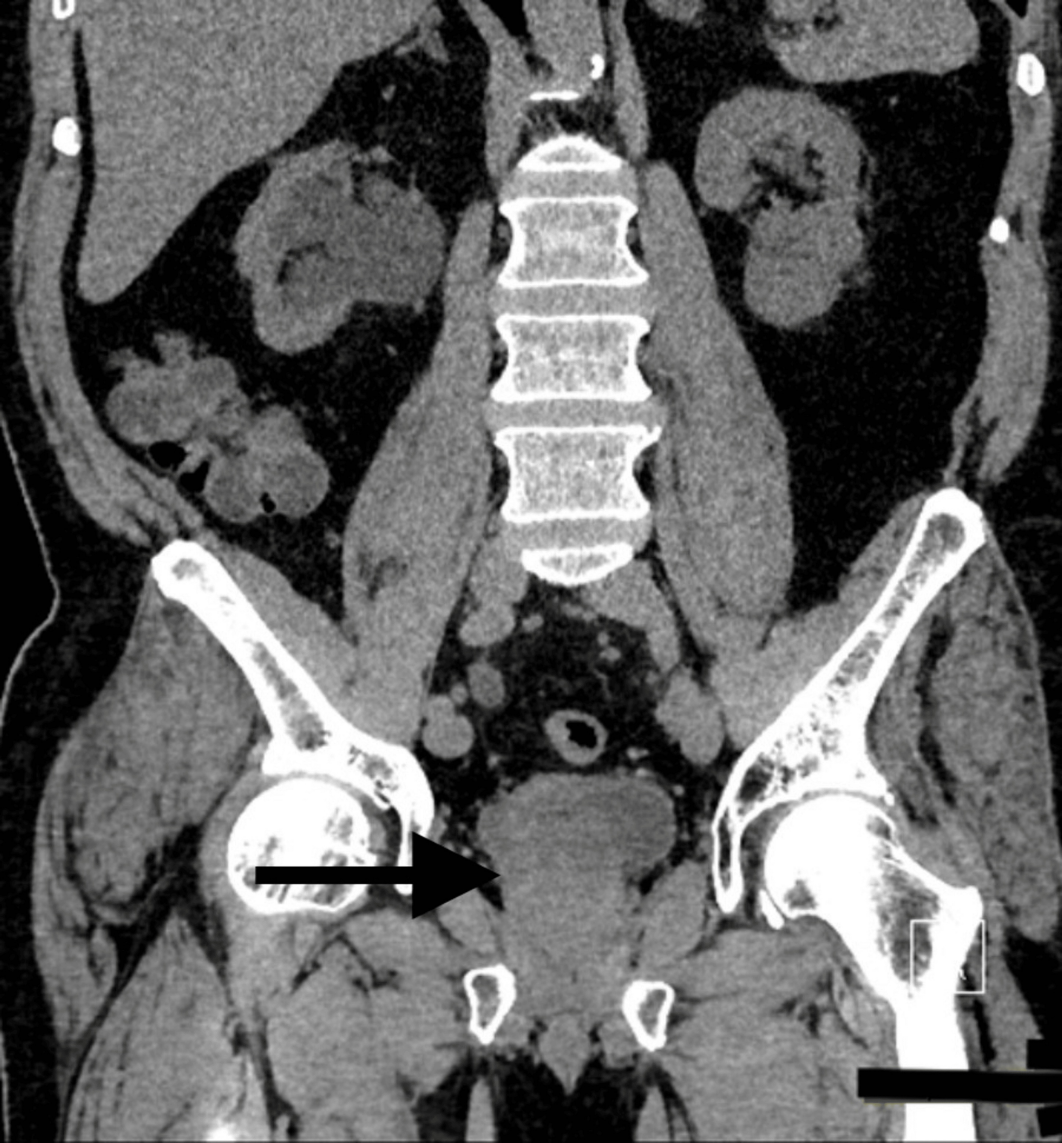
CT abdomen and pelvis coronal view showing bladder mass

During this time, the patient was also complaining of difficulty urination, increased urinary frequency, low back pain, and increased reliance on his catheter. He was then evaluated by the oncologist and bone scintigraphy was obtained, which demonstrated extensive osseous metastases. Additionally, a CT chest abdomen and pelvis were obtained, which demonstrated posterior bladder wall thickening with an exophytic mass abutting the prostate and rectum consistent with neoplasm along with extensive retroperitoneal lymphadenopathy consistent with metastatic disease (Figure 6). He also presented with hydronephrosis and elevated creatinine secondary to ureteral obstruction in the setting of exophytic bladder mass and significant retroperitoneal lymphadenopathy.

**Figure 7:**
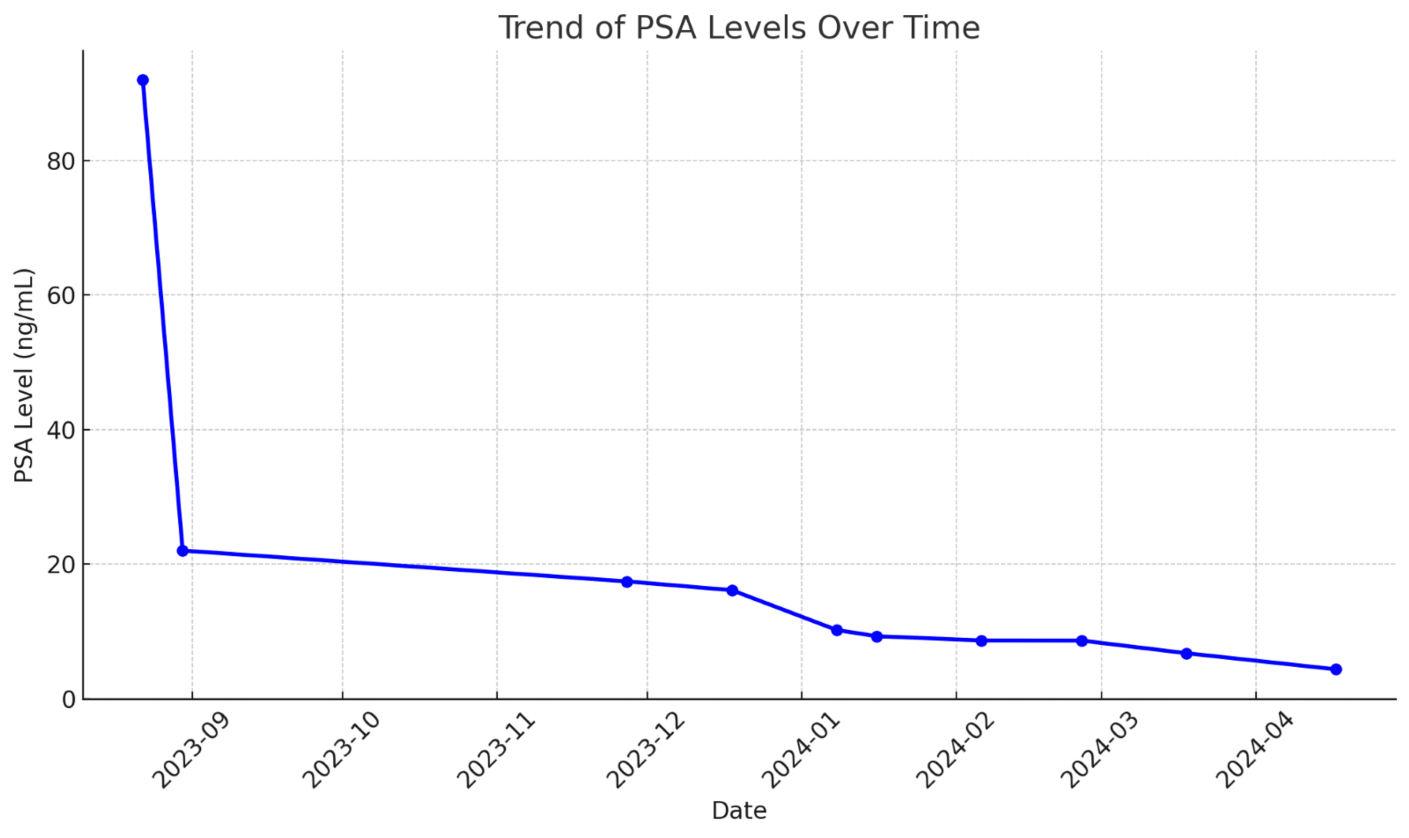
Trend of PSA levels over time PSA reference range – <4 ng/mL is normal PSA: Prostate-specific antigen

**Figure 8:**
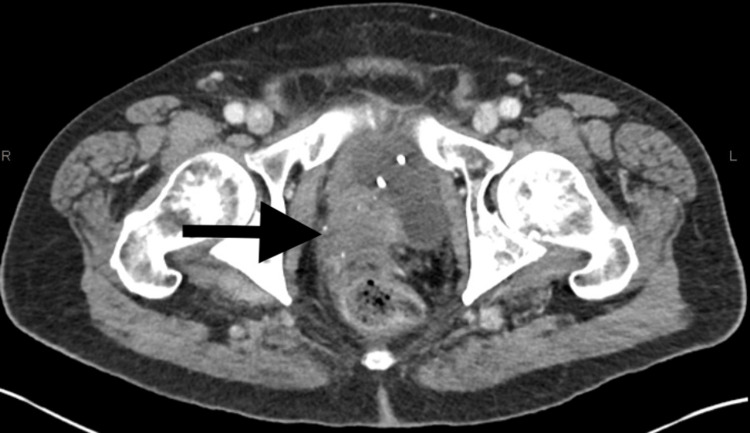
CT abdomen and pelvis axial view (post treatment)

**Figure 9:**
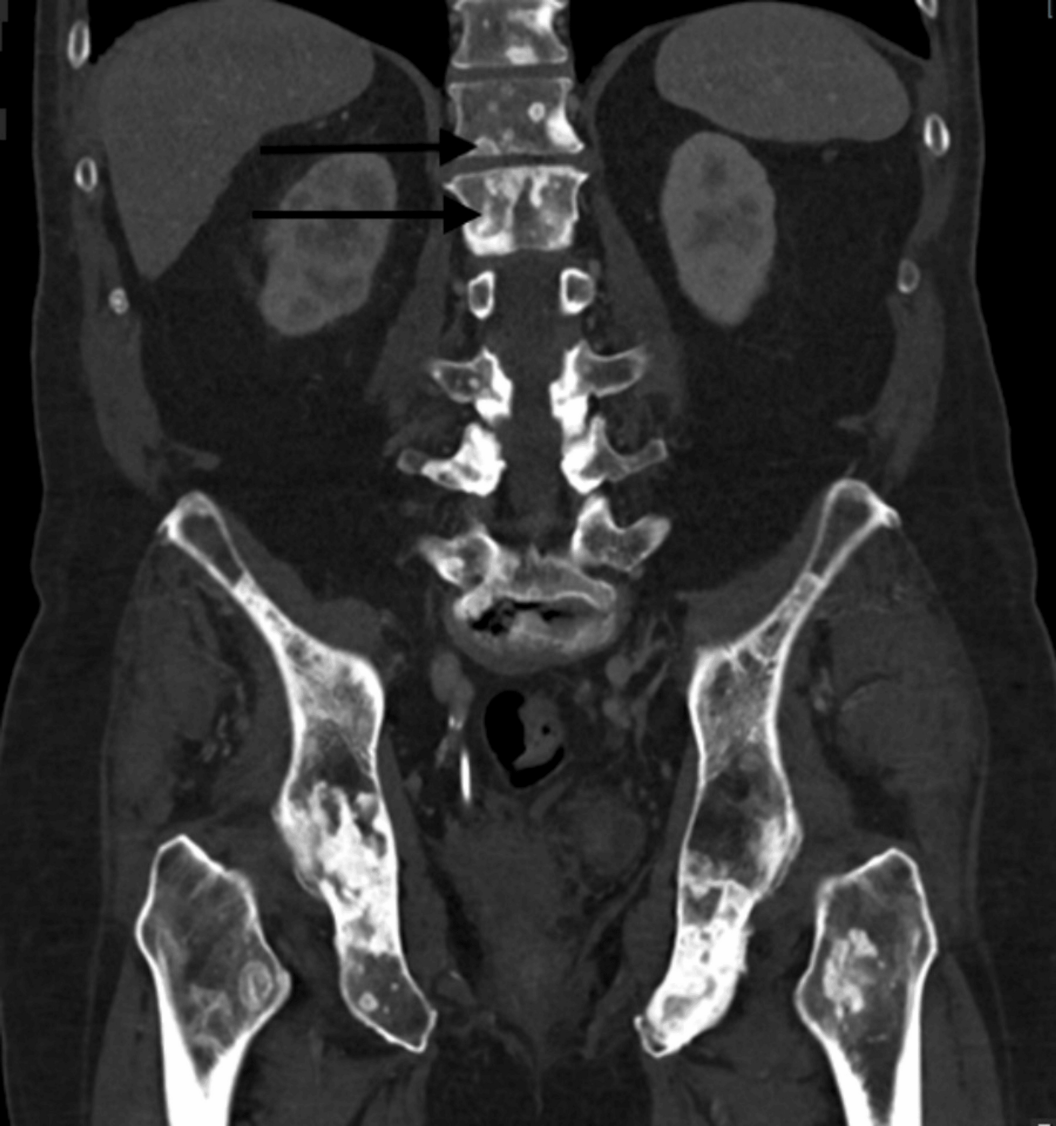
CT showing multiple osseous metastases in the coronal view

**Figure 10:**
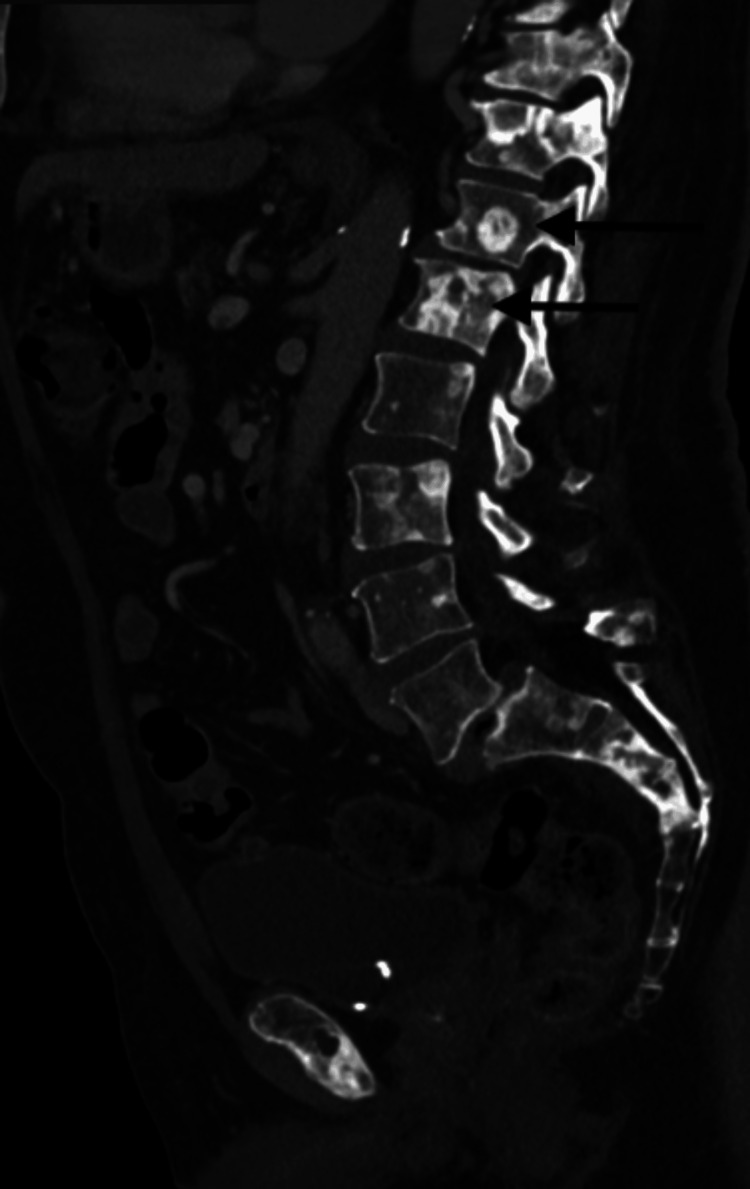
CT showing multiple osseous metastases in the sagittal view

He was diagnosed with metastatic prostate adenocarcinoma, castration naïve, high tumor burden disease with both visceral crisis due to symptomatic left frontal tumor extending into the orbits and osseous involvement. His PSA level at diagnosis was noted to be 92. As per the ARASENS trial, he was then started on combination therapy with a triple regimen including androgen deprivation therapy (ADT), docetaxal, and darolutamide, which demonstrated a significantly improved overall survival rate [10]. He was treated with docetaxel (75mg/m^2^ of body surface area on Day 1 and every 21 days) for six cycles with darolutamide at a dose of 600mg twice daily in addition to relugolix. He was also started on prednisone 5mg BID which will be continued during the therapy. He was noted to have a significant decrease in PSA levels and repeat was 22 after one cycle and then eventually went down to 8.62 (Figure 7). He was also started on denosumab due to extensive bone involvement. He was evaluated by radiation oncology and due to tumor size, location as it is in close proximity to the left optic nerve and orbit, it would not be safe to pursue Gamma knife radiation to the resection cavity so instead, conventional palliative radiation to the surgical bed with 30Gy in 10 fractions was done. Repeat imaging was performed after five months for other reasons due to concern of sepsis and CT abdomen showed interval significant improvement with resolution of abdominopelvic lymphadenopathy (Figure 8). Diffuse osseous metastases and the pelvic mass from the prostate gland which was invading the bladder wall still persists (Figures 9, 10). The treatment was tolerated well except for the onset of diarrhea which was controlled with immodium. He was also referred to cancer genetics for germline testing to assess for BRCA mutation. 

The trend line graph shows the PSA levels over time (Figure 7). It illustrates the significant decline in PSA levels following the initiation of treatment, highlighting the effectiveness of the therapy in reducing PSA over the course of several months.

After completing six cycles of treatment, he was continued on denosumab due to extensive bone involvement. 

Repeat CT chest, abdomen and pelvis were done and showed worsened diffuse sclerotic osseous metastatic disease, particularly in the sternum and thoracic spine, without evidence of a new, displaced pathologic fracture. Given persistent osseous disease, he was started on abiraterone and prednisone, along with denosumab for skeletal bone support.

## Discussion

PCa, though not typically prone to widespread metastasis, most commonly spreads to bones and lymph nodes [6,11]. Atypical metastasis could be seen in PCa and it could be the only presenting symptom. A recent case in 2022 highlighted the potential for misdiagnosis of PCa with metastasis to the lungs given the patient's risk factors of pulmonary disease [12]. Although PCa is typically diagnosed from the prostate or surrounding tissues, about 16% of PCa cases spread to distant organs [13]. However, atypical metastasis, including orbital involvement, has been increasingly reported in rare cases [14]. Due to improved quality of life and decreased mortality rates in patients living with metastatic cancer, the unusual site of spread of malignancy has been increasing [15]. In a study of 508 patients with prostate carcinoma, CT imaging showed that 23 of the patients had atypical nodal metastases, of which 19 of them had unusual spread and 11 had orbital involvement [16]. Primary orbital malignancy is rare but lymphoproliferative lesions are seen in about 20% of orbital masses [17]. In a retrospective case series of 268 patients with various types of orbital lesions, 10% of the orbital tumors were found to be metastatic [18]. The most common primary tumor site for orbital metastasis is breast cancer which is about 53%, followed by the prostate gland with a risk of 12% [19]. In a retrospective chart review it was found that the most frequent primary tumors of uveal metastases were breast (36.3%), melanoma (10.1%), and prostate (8.5%) cancers [8]. 

Orbital metastasis from PCa is uncommon but presents unique diagnostic and management challenges. Patients with orbital metastases generally have a poor prognosis, with a median survival of 1.3 years [9]. This highlights the importance of early identification and intervention. The most common symptoms of orbital metastatic disease include blurred vision, diplopia, pain, and a lump beneath the eyelid. Clinical signs such as limited ocular motility (54%), proptosis (50%), and palpable masses (43%) are frequently noted on examination [20]. Advanced imaging techniques, particularly MRI of the brain and orbits, play a crucial role in identifying these lesions [9]. Histopathological examination and immunohistochemistry (e.g., NKX3.1 nuclear staining) are pivotal for confirming the prostatic origin of metastatic lesions [20]. 

Multimodal treatment approaches, as recommended by the National Comprehensive Cancer Network (NCCN), have been shown to improve outcomes. The treatment options are determined based on the nature of the tumor and severity of the disease and include chemotherapy, radiation therapy, hormonal therapy, surgery, cryotherapy, and active surveillance. To determine the clinical stage of PCa, multiparametric MRI and DRE should be performed. The NCCN and the European Association of Urology-European Society for Radiotherapy and Oncology-International Society of Geriatric Oncology (EAU-ESTRO-SIOG) favor multimodal treatment approaches such as radical prostatectomy with adjuvant radiotherapy or a combination of radiotherapy and androgen deprivation therapy (ADT) [21]. To eradicate the tumor both locally and microscopically, this multimodality approach is the ideal treatment [22]. Surgical resection, when feasible, remains a cornerstone of managing orbital metastases, especially when combined with radiotherapy [20]. In cases where surgery is contraindicated due to tumor location or patient health, palliative radiotherapy may provide symptomatic relief and improve quality of life [9].

A recent systematic review of orbital metastases by Palmisciano and colleagues highlights that the metastatic disease may significantly worsen the functional status of oncological patients, leading to debilitating visual impairments. Additionally, the data showed that surgical resection and orbital radiotherapy were related to improved clinical outcomes [9]. For this reason, ophthalmological intervention should be considered for treatment and management regarding orbital involvement of metastatic disease. 

The management of PCa with atypical metastasis should also integrate systemic therapies tailored to the disease’s burden and patient-specific factors. For this patient, a combination regimen including ADT, docetaxel, and darolutamide, as per ARASENS trial recommendations, demonstrated significant efficacy in reducing PSA levels and improving survival rates [10]. Additionally, denosumab was incorporated to address extensive osseous involvement, demonstrating the importance of a multidisciplinary approach.

Emerging evidence also emphasizes genetic evaluation for germline mutations, such as BRCA, which may inform targeted therapies and further personalize treatment [6]. As this case illustrates, an integrated approach that includes oncological, ophthalmological, and palliative care components can optimize outcomes, even in rare presentations like orbital metastases.

## Conclusions

In conclusion, this case highlights the rare presentation of orbital metastasis from prostate adenocarcinoma, underscoring the necessity for a high index of suspicion in similar clinical scenarios. Thorough ophthalmological and systemic workups, including imaging and histopathological evaluation, are critical for accurate diagnosis. Accurate diagnosis, through comprehensive imaging and immuno-histochemical testing, proved essential in guiding appropriate management, including surgical resection, radiation, and systemic therapies. A multidisciplinary, evidence-based management approach integrating surgical resection, radiotherapy, and systemic therapy significantly contributes to improved clinical and functional outcomes. While rare, metastatic PCa should remain a differential consideration in cases of orbital tumors, emphasizing the importance of individualized and comprehensive care strategies in managing atypical metastatic disease. Further studies are warranted to better understand and manage these unusual metastatic patterns in PCa, particularly given their association with poor prognosis and challenging symptomatology. 

## References

[1] Rawla P (2019). Epidemiology of prostate cancer. World J Oncol.

[2] Clark R, Herrera-Caceres J, Kenk M, Fleshner N (2022). Clinical management of prostate cancer in high-risk genetic mutation carriers. Cancers (Basel).

[3] Miller KD, Nogueira L, Devasia T (2022). Cancer treatment and survivorship statistics, 2022. CA Cancer J Clin.

[4] Sekhoacha M, Riet K, Motloung P, Gumenku L, Adegoke A, Mashele S (2022). Prostate cancer review: genetics, diagnosis, treatment options, and alternative approaches. Molecules.

[5] Vietri MT, D'Elia G, Caliendo G (2021). Hereditary prostate cancer: genes related, target therapy and prevention. Int J Mol Sci.

[6] Gandaglia G, Abdollah F, Schiffmann J (2014). Distribution of metastatic sites in patients with prostate cancer: a population-based analysis. Prostate.

[7] Bubendorf L, Schöpfer A, Wagner U (2000). Metastatic patterns of prostate cancer: an autopsy study of 1,589 patients. Hum Pathol.

[8] Shields CL, Shields JA, Gross NE, Schwartz GP, Lally SE (1997). Survey of 520 eyes with uveal metastases. Ophthalmology.

[9] Palmisciano P, Ferini G, Ogasawara C (2021). Orbital metastases: a systematic review of clinical characteristics, management strategies, and treatment outcomes. Cancers (Basel).

[10] Smith MR, Hussain M, Saad F (2022). Darolutamide and survival in metastatic, hormone-sensitive prostate cancer: a patient and caregiver perspective and plain language summary of the ARASENS trial. Future Oncol.

[11] Manna F, Karkampouna S, Zoni E, De Menna M, Hensel J, Thalmann GN, Kruithof-de Julio M (2019). Metastases in prostate cancer. Cold Spring Harb Perspect Med.

[12] Lyonga Ngonge A, Amadife SN, Wireko FW, Ikwu I, Poddar V (2022). A case report on atypical presentation of metastatic prostate cancer. Cureus.

[13] Klusa D, Lohaus F, Furesi G (2020). Metastatic spread in prostate cancer patients influencing radiotherapy response. Front Oncol.

[14] Allen RC (2018). Orbital metastases: when to suspect? when to biopsy?. Middle East Afr J Ophthalmol.

[15] Di Micco R, Santurro L, Gasparri ML (2019). Rare sites of breast cancer metastasis: a review. Transl Cancer Res.

[16] Long MA, Husband JE (1999). Features of unusual metastases from prostate cancer. Br J Radiol.

[17] Laplant J, Cockerham K (2021). Primary malignant orbital tumors. J Neurol Surg B Skull Base.

[18] Shinder R, Al-Zubidi N, Esmaeli B (2011). Survey of orbital tumors at a comprehensive cancer center in the United States. Head Neck.

[19] Shields JA, Shields CL, Brotman HK, Carvalho C, Perez N, Eagle Jr RC (2001). Cancer metastatic to the orbit: the 2000 Robert M. Curts Lecture. Ophthalmic Plast Reconstr Surg.

[20] Shields JA, Shields CL, Brotman HK, Carvalho C, Perez N, Eagle Jr RC (2012). Cancer metastatic to the orbit: The 2012 Zimmerman lecture. Ophthalmic Plast Reconstr Surg.

[21] Faiena I, Kim IY, Jang TL (2019). Multimodal treatments for advanced prostate cancer. Oncotarget.

[22] Terlizzi M, Bossi A (2022). High-risk locally advanced prostate cancer: multimodal treatment is the key. Eur Urol Open Sci.

